# A novel insertion-induced frameshift mutation of the androgen receptor gene in a patient with primary amenorrhea^☆^

**DOI:** 10.1016/j.mgene.2013.10.011

**Published:** 2013-11-28

**Authors:** Vikas Sharma, Kumarasamy Thangaraj, Akka Jyothy

**Affiliations:** a Department of Biotechnology, University College of Science, Osmania University, Hyderabad, India; b Centre for Cellular and Molecular Biology, Hyderabad, India; c Institute of Genetics and Hospital for Genetic Diseases, Osmania University, Hyderabad, India

**Keywords:** AR, androgen receptor, SRY, sex-determining region Y, PCR, polymerase chain reaction, LBD, ligand-binding domain., AR gene, Primary amenorrhea, Novel mutation

## Abstract

**Objective:**

To report a novel single nucleotide insertion mutation, and present the clinical, genetic, biochemical findings in a patient with primary amenorrhea.

**Methods:**

Chromosomal analysis was performed by harvesting lymphocytes from peripheral blood sample. Hormonal analysis was performed from the serum. After genomic DNA extraction from peripheral blood leukocytes the coding regions and corresponding exon–intron boundaries of sex-determining region Y (SRY) gene and androgen receptor (AR) gene were amplified by PCR and subjected to direct sequencing.

**Results:**

In the patient with a karyotype 46,XX, we identified a novel single nucleotide insertion mutation of the nucleotide G at position 2369 (GenBank accession number HM010955), resulting in amino acid interchange cysteine to tryptophan at codon 669 in exon 4 [Cys669Trp] (GenBank Protein_id ADF47187).

**Conclusions:**

We report a novel single nucleotide insertion mutation in exon 4 region of the AR gene. The nature of the mutation presented in the patient is in the ligand-binding domain (LBD) of the AR gene. This insertion mutation was predicted to produce frame shift mutation and resulted in truncated form of the AR protein, implicating it in the phenotype observed with primary amenorrhea.

## Highlights

• AR is a member of the steroid receptor superfamily.
• AR gene was screened in a patient with primary amenorrhea by direct PCR sequencing.
• A novel insertion mutation (c.2369_2370insG, p.Cys669TrpfsX12) was identified.
• This causes frame shift mutation resulting in truncated form of the AR protein.
• The finding further enriched the AR mutation spectrum.

## Introduction

The androgen receptor (AR) is a member of the steroid receptor superfamily and involved in the process of sex differentiation. The principle action of androgen is to regulate gene expression through the AR. Mutations in the AR have been correlated with a wide spectrum of androgen insensitivity syndromes (AIS), that span from mild androgen insensitivity syndrome (MAIS) and partial androgen insensitivity syndrome (PAIS) to complete androgen insensitivity syndrome (CAIS). The symptoms range from phenotypically normal male with impaired spermatogenesis to phenotypically normal women with primary amenorrhea.

AR has a well characterized modulator structure and is organized into functional domains. The AR gene is located on chromosome Xq11-12. Spanning 90 kb, it comprises 8 exons with 2757 bp of open reading frame within a 10.5-kb messenger RNA (mRNA) encoding for a modular protein of 919 amino acid residues (Tilley et al., 1989), with three functional domains. The N-terminal domain (NBD) is encoded by exon 1, the DNA-binding domain (DBD) is encoded by exons 2 and 3, and the C-terminal ligand-binding domain (LBD) is encoded by five exons 4–8 (Lubahn et al., 1988). More than 500 AR gene mutations have been reported in the recent past among AIS patients, indicating a great deal of genetic heterogeneity (Gottlieb et al., 2004).

The N-terminal transactivation domain is the largest AR domain, but it contains less than 15% of AR mutations leading to androgen insensitive syndrome (Radpour et al., 2009). The C-terminal region comprises the DNA-binding domain and ligand-binding domain, which contain about 20% and 60% of the mutations identified, respectively (Gelmann, 2002). A large number of AR mutations are caused by single nucleotide substitutions or deletions.

We present a case of young woman affected with primary amenorrhea. The sequencing of AR gene revealed the presence of a novel insertion mutation that created a subsequent frame-shift mutation and resulted in truncated form of AR.

## Case report

A 19-year-old girl was referred to our institute because of primary amenorrhea, poor breast development, tall stature and without history of previous illness. She is the first daughter born to nonconsanguineous healthy parents. Her mother's pregnancy and delivery were normal. There was no family history of delayed puberty or infertility. At this stage the girl was screened for the clinical, genetic, biochemical, and molecular investigations. The study was approved by the institutional ethics committee, and informed written consent was obtained from the subject.

The girl was 175 cm tall and weighed 55 kg. She showed normal body mass index, normal IQ and absence of Turner's stigma. The physical examination showed poor pubertal development (breast, Tanner stage II; pubic hairs, Tanner stage III) with no virilization. The clinical examination of the genitals showed normal female genitalia, clitoris and partially fused labia. Abdominal and retroperitoneal ultrasonographic studies detected hypoplastic uterus and absence of ovaries. Genitogram studies revealed blind-ending vagina and absence of cervix.

## Materials and methods

### Hormonal assay

Serum testosterone (T), follicle-stimulating hormone (FSH), and luteinizing hormone (LH) levels were measured by radioimmunoassay.

### Cytogenetic studies

Chromosome analysis was performed by lymphocyte culture obtained from the peripheral blood of the subject. Conventional staining and the standard GTG banding technique were applied (Seabright, 1971). The karyotype analysis was performed with Leica CW4000 Karyo imaging software (Leica Imaging Systems, Cambridge, United Kingdom).

### DNA sequence analysis

For mutational analysis, Genomic DNA was extracted from peripheral blood samples of the subject (Thangaraj et al., 2002). The SRY gene amplification and sequencing were performed using specific primers. The AR coding regions and flanking intronic sequences of exons 2–8 and nonpolymorphic regions of exon 1 were also amplified by polymerase chain reaction (PCR) by the use of primers listed in Table 1. The 10 μL of PCR reaction mixture included 1.0 μL of PCR buffer (10 ×), 1.0 μL of MgCl2 (25 mM), 0.8 μL of deoxynucleotide triphosphates (10 mM), 1 pM of each primer, 1 unit of AmpliTaq Gold DNA polymerase (Applied Biosystems, Foster City, CA), and 20 ng of genomic DNA.

The SRY gene PCR conditions were initial denaturation at 94 °C for 1 min followed by 30 cycles of 94 °C for 30 s, 65 °C for 30 s, 72 °C for 1 min, with final extension at 72 °C for 5 min. The PCR conditions for exon 4 of the AR gene consisted of initial denaturation at 94 °C for 12 min followed by 30 cycles of denaturation at 94 °C for 45 s, annealing at 58 °C for 45 s, and an extension at 72 °C for 1 min, with a final extension at 72 °C for 10 min. The DNA sequencing was performed with the PCR primers using the BigDye terminator v3.1 Cycle Sequencing Kit and DNA sequences run on an ABI Prism 3100 Genetic Analyzer (Applied Biosystems). All the remaining exons of the AR gene were directly sequenced to confirm the presence of any other mutation.

## Results

**Fig. 1:**
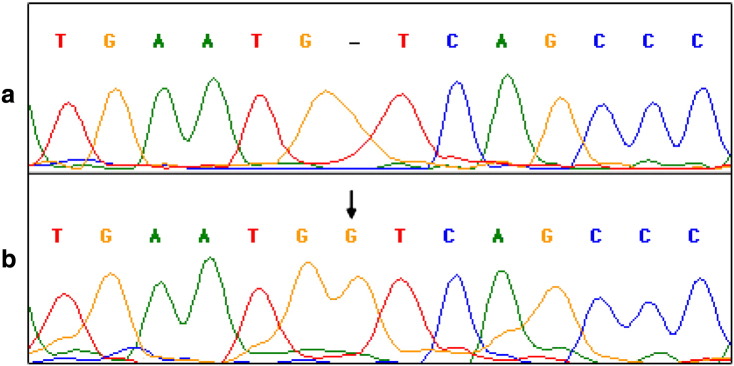
Sequence analysis of the androgen receptor gene mutation. DNA sequence of exon 4 region of AR from a control individual (a) and the patient (b). The sequencing result of the patient (b) shows insertion of nucleotide G indicated by the arrow.

The hormone profile indicated the concentration of T, FSH and LH as 0.30 ng/mL, 4.30 mIU/mL and 1.97 mIU/mL respectively. The screening of 200 metaphases for chromosomal analysis revealed 46,XX karyotype. The SRY gene amplification was performed using specific primers and the subject was found to be SRY negative. All exons of AR gene were successfully amplified, and sequence analysis revealed a novel single nucleotide G (Guanine) insertion at nucleotide number 2369 (GenBank accession number: HM010955). The mutation was identified in the exon 4 region of AR gene that changes the sense of codon 669 from TGT (Cysteine) to TGG (Tryptophan) (GenBank Protein_id ADF47187). This mutation results in a frameshift and created a termination codon in place of His681X that resulted in a truncated form of AR protein. The mutation was confirmed by bidirectional sequencing (Fig. 1).

## Discussion

Mutations in the AR gene are reported with a wide spectrum of clinical manifestations. In the present subject, a novel insertion mutation was identified in the exon 4 region of AR gene. The diagnosis was made on the basis of the clinical, cytogenetic, biochemical, and molecular analysis.

The LBD of AR protein is encoded by the exon 4 to 8. This domain represents a region of highest homology among the nuclear receptors. The functions of the domain include heat shock protein interactions, dimerization and nuclear localization signals (Hughes et al., 2003). Numerous mutations have been identified, that are clustered throughout the AR protein coding region. These mutations are known to show phenotypic variation in different individuals. The Exon 4 constitutes the essential part of the LBD, with reports of more than 100 mutations. Most of these reported mutations are substitution mutation and only two are insertion mutations. None of the mutation is reported at codon 669. The novel insertion mutation (c.2369_2370insG, p.Cys669TrpfsX12) introduces a premature termination codon at amino acid His681X and resulted in a truncated form of the receptor, devoid of 238 carboxy-terminal amino acids, including a major part of LBD and the region responsible for dimerization and ligand-dependent transactivation.

## Conclusions

On the basis of DNA analysis we conclude that the novel insertion mutation (c.2369_2370insG), results in amino acid change Cys669Trp in the conserved LBD of AR. This mutation results in a frameshift and created a termination codon in place of His681X that resulted in a truncated form of the AR protein, critical for AR function. This might result in a decrease in or even abolish the ligand-dependent transactivation. Thus, the novel insertion mutation identified in the AR gene is responsible for the symptoms in the subject and this finding is consistent with the clinical diagnosis. To the best of our knowledge, the mutation described in our study is novel and previously unpublished.

## Figures and Tables

**Table 1:** List of PCR primers used for amplifying exons and flanking intron regions of AR gene.

Exon no.	Primer sequence	Product size (bp)
1	AAGAGAAGGGGAGGCGGGGTAAG CAACGTGGATGGGGCAGCTGAG	521–584
1	CCCGAGAGAGGTTGCGTCCC TCGCCTTCTAGCCCTTTGGTG	592
1	AGCTTCGGGGGGATTGCATGTA GCCGCCAGGGTACCACACATC	739
1	CCGCTTCCTCATCCTGGCACAC TCTGAAGGTGGCCCGTGCAATAG	617–668
2	AATGCTGAAGACCTGAGACT AAAATCCTGGGCCCTGAAAG	318
3	CTAGAAATACCCGAAGAAAG GAGAGACTAGAAAATGAGGG	257
4	GTGATTTTCTTAGCTAGGGC ATCCCCCTTATCTCATGCTC	424
5	GCTTTTCCCCACCACCCCTTAAT TTGGGTGTGAAAGGGGTGGTCTC	527
6	CCAGCAGGAGAAACAGCAAGC GGGGAATGAAGAAGGGAAATGTC	378
7	AGGCCCCAAGCACACAGACT CCTCCACCCCTTTCACAATATC	516
8	GCCACCTCCTTGTCAACCCT AGAGGAGTAGTGCAGAGTTA	289
